# The inherence bias in preschoolers’ explanations for achievement differences: replication and extension

**DOI:** 10.1038/s41539-024-00218-w

**Published:** 2024-02-20

**Authors:** Margaux Renoux, Sébastien Goudeau, Theodore Alexopoulos, Cédric A. Bouquet, Andrei Cimpian

**Affiliations:** 1 Centre de Recherches sur la Cognition et l’Apprentissage (UMR 7295), Université de Poitiers, CNRS, Poitiers, France; 2 Laboratoire de Psychologie (UR4139), Université de Bordeaux, Bordeaux, France; 3 Laboratoire de Psychologie Sociale et Cognitive (UMR 6024), Université Clermont Auvergne, CNRS, Clermont-Ferrand, France; 4 Department of Psychology, New York University, New York, NY USA

**Keywords:** Social sciences, Psychology

## Abstract

Two studies examined how preschoolers (*N* = 610; French) explain differences in achievement. Replicating and extending previous research, the results revealed that children invoke more inherent factors (e.g., intelligence) than extrinsic factors (e.g., access to educational resources) when explaining why some children do better in school than others. This inherence bias in explanation can contribute to inequalities in education (e.g., the early-emerging disparities based on social class) by portraying them as fair and legitimate even when they are not.

## Introduction

Even at the earliest stages of schooling, such as preschool, children pay attention to how well their peers do academically and try to explain the differences between them^1,2^. The key hypothesis tested in the present work is that when preschool children explain the achievement differences they observe in the classroom, they invoke factors inherent to students (e.g., intelligence, personality) more often than is justified and, conversely, overlook factors extrinsic to students (e.g., family background^3^). In the spirit of contributing to cumulative and reproducible science^4^, we replicate and extend an important set of prior findings that provided support for this hypothesis, suggesting that preschool children indeed show an *inherence bias* in their explanations for achievement differences in the classroom.

Essentially, the inherence bias^5–7^ is a general version of the so-called correspondence bias documented in social psychology, whereby a person’s actions are often assumed to correspond to their internal dispositions (for a review, see ref. ^8^). The bias to rely on inherent factors and overlook extrinsic factors when generating explanations applies to a wide range of observations—not just behavior. For example, the inherence bias has been documented in explanations for social conventions^9,10^, historical events^11^, features of language^12^, and natural phenomena^11^. This explanatory bias also contributes to the development of psychological essentialism—the belief that there are internal “essences” that differentiate (some) social groups from each other and that cause members of these groups to think and behave in particular ways (e.g., refs. ^13,14^).

The inherence bias arises from a combination of cognitive factors, including attentional spotlight effects (when seeking explanations, people tend to focus on the central figure and often overlook context), accessibility differences in long-term memory (inherent properties are easier to recall from long-term memory than contextual factors), and working memory limitations (inherent properties are simpler and easier to manipulate given the constraints of working memory; for a review, see ref. ^7^). The effects of these cognitive factors are amplified or attenuated by the cultural context, which can, for example, broaden or narrow the scope of attention or make certain extrinsic information more accessible in long-term memory than it would otherwise be. In Western contexts, for instance, the pervasive idea that individuals are independent, autonomous agents separated from their social context^15^ may amplify the tendency to explain a person’s behavior by appealing to their inherent characteristics^16,17^.

To clarify, the term “bias” here is meant to indicate that everyday explanations deviate systematically from those that a perfectly rational thinker would generate. In our usage, this term does not denote an inescapable, deterministic pattern of explanation. In fact, it is an explicit part of the theory that the degree of deviation from the standards of a perfectly rational and unbiased thinker will *vary* from case to case depending on the reasoner, the context, and the observation being explained^5–7^.

The inherence bias in explanation is of interest to scholars of inequality because this bias often leads people to accept the inequalities they observe around them, making these inequalities seem “natural” and unchangeable^18–20^. For example, 4- to 8-year-old children who were told about status disparities between fictional groups on “a planet far, far away” explained these disparities predominantly in terms of inherent features (e.g., the higher-status group is smarter and harder-working^18^). In turn, children who adopted inherent explanations were also more likely to think that the status disparities were “fair” and “okay.” Extending this argument to inequality in education, it is possible that the inequalities that arise early on between, for example, children from working-class backgrounds and their peers from middle- and upper-class backgrounds^21^ are similarly interpreted as a product of the inherent characteristics of children from these groups.

In addition to legitimizing these inequalities, which are in reality unfair and illegitimate, inherent explanations could have implications for how children from the relevant groups perceive themselves and are perceived by others. In light of these explanations, children who do well in school may be perceived as inherently more worthy (e.g., smarter, harder-working), whereas the opposite would hold for children who encounter difficulties in school—even when these difficulties are not, in fact, a reflection of any personal shortcomings. The problematic nature of these inferences is especially salient when we consider that children from marginalized backgrounds, such as those from working-class or ethnic-minority communities, are more likely to experience difficulty in the classroom^21^. Explaining these differences in inherent terms is likely to lower the self-concepts and self-efficacy of children from marginalized backgrounds^22,23^. Social class and racial/ethnic differences in self-perceptions and perceptions by others could then act as a self-fulfilling prophecy, amplifying initial inequalities in achievement^24,25^.

Consistent with the claim that the inherence bias legitimizes inequality, Goudeau and colleagues^26^ found that preschool children tend to (a) explain differences among their peers in how much they contribute to whole-class discussions by invoking inherent factors (e.g., intelligence, effort) rather than extrinsic factors (e.g., family background) and (b) perceive peers who contribute more to whole-class discussions as more competent and warmer. This finding is relevant to inequality in education because, as early as preschool, children from working-class families are less likely to speak during whole-class discussions, and when they do, they speak less compared to their middle- and upper-class peers^26^ (see also refs. ^27–30^). Goudeau et al. ^26^ went beyond simply documenting these differences to show that when preschool children explained the differences in participation they observed among their peers, they tended to appeal to inherent attributes, assigning higher competence and warmth to peers who contribute more than others. To the extent that children from middle- and upper-class families are those who contribute most during classroom discussions, these positive perceptions of competence and warmth could legitimize, and perhaps even amplify, academic inequalities between children of different social classes^31–34^.

In Study 1 (preregistered), we tested whether Goudeau and colleagues’^26^ finding of an inherence bias in children’s explanations for differences in contributions to whole-class discussions replicates in a different, larger sample (see Hypotheses 1 and 2 in Table 1). An ancillary goal of Study 1 was to examine whether children accurately perceive social class differences in contributions^26^. We expected that children from working-class (vs. middle- and upper-class) backgrounds would perceive that they contribute less to whole-class discussions (Hypothesis 3). We also expected that children would perceive their working-class (vs. middle- and upper-class) peers as contributing less to whole-class discussions (Hypothesis 4). A final ancillary hypothesis, Hypothesis 5, examined the possibility of response biases and is detailed below.

**Table 1:** Full list of preregistered hypotheses for Study 1

Hypothesis number	Description of hypothesis
1	Children will use more inherent (vs. extrinsic) factors to explain why a fictional child makes particularly frequent or long contributions to oral exchanges in the classroom.
2	Children will judge the fictional child as higher in competence and warmth than other children in their class.
3	Children from working-class backgrounds will be less likely than children from middle- and upper-class backgrounds to perceive themselves as similar to the fictional child.
4	Children from working-class backgrounds will be less likely than children from middle- and upper-class backgrounds to be perceived by their peers as similar to the fictional child.
5	Children’s answers to a control question will not differ significantly from the midpoint of the scale, speaking against the possibility of response biases.

The language was adapted slightly from the preregistration to fit the terminology used in the present manuscript. These changes were superficial and did not affect the content of the hypotheses. Hypotheses 3–5 were not tested in the original Goudeau et al.^26^ study and are thus labeled “ancillary”.

In Study 2, we further probed the robustness of the hypothesized inherence bias in explanations for achievement differences. Specifically, we examined whether this explanatory bias is observed when children explain differences in achievement more broadly, beyond oral participation. We also examined whether the bias persists when children explain differences in achievement between themselves and their peers, as opposed to differences between fictional children, as in Study 1.

## Results

### Study 1

Participants included 306 preschoolers (enrolled with administrative authorization and written parental consent) from 27 classrooms of *Grande-Section*, the last year in French preschool, roughly equivalent to kindergarten in the USA and other countries (142 girls, 164 boys; *M*_age_ = 5.6 years, *range* = 4.9–6.6).

Although information about the ethnicity of the children in our sample was not available (because this information cannot be collected in France due to current laws), the children were recruited from a region of France where the population is ethnically homogeneous—mostly white^35^.

We used the Social Position Index as a proxy for social class^36^. This indicator is a standardized continuous variable with a mean of 100 and a standard deviation of 30. It has been developed on large French databases in order to capture multiple dimensions linked to social class (e.g., educational attainment, parental education, material conditions, cultural capital). We collected information about the occupation of each parent and then assigned a Social Position Index value to each child based on these occupations^36^. The composition of this study’s sample in terms of social class was similar to the social structure of French society (*M* = 109, *SD* = 26.1). For analysis purposes, we divided the sample based on a median split (*Mdn* = 106.5). We referred to the two groups that resulted from this split as “working class” (*N* = 150, 49%) and “middle and upper class” (*N* = 156, 51%).

Following a power analysis based on the effect size from Goudeau and colleagues’ original study^26^, we preregistered a sample of 200 children. To compensate for potential logistical issues caused by the COVID crisis, we increased the number of classrooms we contacted. Unexpectedly high rates of interest from teachers and parents resulted in a final sample of 306 participants. A sensitivity analysis using this larger sample indicated that we had 80% power to detect effects of magnitude *w* ≥ 0.16 on a χ² test and *d* ≥ 0.16 on a one-sample *t* test.

Children were tested individually in a quiet room next to their classroom. The sessions lasted around 10 minutes and were recorded and later transcribed. Below, we first describe the measures that map directly onto Goudeau and colleagues’^26^ study, which are directly relevant to our goal of testing whether we can replicate their results (see Hypotheses 1 and 2 in Table 1). Then, we describe additional measures that were added to examine ancillary hypotheses that were not investigated in the original study (see Hypotheses 3–5 in Table 1).

#### Measure: open-ended explanations (Hypothesis 1)

Children were read two hypothetical scenarios describing children participating in whole-class discussions in preschool. The first scenario focused on the *frequency* of participation and described a situation in which the teacher calls on a child more often than other children (e.g., “When the teacher asks a question to the class, several children raise their hands. However, the teacher calls on [Theodore/Zélie] more often than other children”). The second scenario focused on the *duration* of participation and described a situation in which a child speaks for a longer period of time than other children (“When the teacher asks a question to the class, several children raise their hands. When the teacher asks [Leopold/Suzon] a question, [Leopold/Suzon] talks longer than the other children”). In France, Theodore and Leopold are typically first names for boys, and Zélie and Suzon are typically first names for girls. The order of presentation of the scenarios and the gender of the protagonist in each scenario were counterbalanced across participants.

Children were asked to explain the achievement-related outcome in each scenario (“Why do you think [Theodore/Zélie] is called on more often than other children?” for Scenario 1 and “Why do you think [Leopold/Suzon] talks longer than the other children?” for Scenario 2). If the children said they did not know, two follow-up questions were asked (e.g., “There is no wrong answer. Do you want to try to guess? Why […]?”). If the child still did not answer after these follow-ups, the experimenter moved on to the next question.

Children’s answers were coded independently by two researchers using four categories: (1) inherent factors (e.g., “because she/he is intelligent”; “because she/he knows a lot of things”; “because she/he is wise”), (2) extrinsic factors (e.g., “because the teacher likes her/him”, “because the other children do not want to talk”; “because she explains something important”), (3) incoherent or irrelevant explanations (e.g., “because she/he is small”; “because it’s the last day before the holidays”), and (4) no explanation (e.g., “I don’t know”). Inter-rater reliability was high (Scenario 1: 96% agreement, Cohen’s κ = 0.92; Scenario 2: 94% agreement, Cohen’s κ = 0.86), and disagreements were resolved through discussion. We also calculated inter-rater reliability after excluding the “no explanation” category (Scenario 1: 94% agreement, Cohen’s κ = 0.82; Scenario 2: 92% agreement, Cohen’s κ = 0.52). The κ value for Scenario 2 was lower than expected but was still “fair” by conventional standards^37^.

#### Measure: competence and warmth evaluations (Hypothesis 2)

After the open-ended explanation questions, children were reminded of each scenario and were invited to assess the protagonists on the two fundamental dimensions of social judgment, competence and warmth^38^, on a scale ranging from 1 (*a lot less than other children*) to 5 (*a lot more than other children*), with a midpoint of 3 (*the same as the other children*). The addition of an explicit midpoint was a methodological improvement over the original study^26^, in which children were forced to decide whether the protagonist was higher or lower than their peers on a particular dimension. Two items assessed perceptions of competence:

1. perceived intelligence (“How intelligent do you think [child] is? Do you think [child] is more intelligent than the other children, as intelligent as the other children, or less intelligent than the other children?”; follow-up: “Do you think [child] is a little [more/less] intelligent, or a lot [more/less] intelligent than the other children?”), and
2. perceived academic achievement (“How good at school do you think [child] is? Do you think [child] is better at school than the other children, as good at school as the other children, or worse at school than the other children?”; follow-up: “Do you think [child] is a little [better/worse] at school, or a lot [better/worse] at school than the other children?”).
  Two other items assessed perceptions of warmth:
3. perceived niceness (“How nice do you think [child] is? Do you think [child] is nicer than the other children, as nice as the other children, or less nice than the other children?”; follow-up: “Do you think [child] is a little [nicer/less nice] or a lot [nicer/less nice] than the other children?”), and
4. teacher’s perceived liking of the protagonist (“How much do you think the teacher likes [child]? Do you think the teacher likes [child] more than the other children, as much as the other children, or less than the other children?”; follow-up: “Do you think the teacher likes [child] a little [more/less] or a lot [more/less] than the other children?”).

To explore potential low-level response biases on this task (e.g., children always choosing the first option or preferring the “more than others” option), we added three control items that were not present in the original study^26^. Specifically, we asked children whether the protagonist was “mean” and “stupid” and whether they “liked strawberries,” which was chosen to be unrelated to school. We expected children to choose the “less than others” option for the two negative items and to be close to the midpoint (“the same as the others”) for the unrelated item (see Hypothesis 5 in Table 1). In contrast, if children chose the first option (“more than others”) across all these items as well, this would suggest a response bias. The presentation order of the seven items (the four original items plus three control items) was counterbalanced across children.

#### Additional measures

Several measures were added to the present replication study to test ancillary hypotheses that had not been investigated by Goudeau and colleagues^26^ (see Hypotheses 3 and 4 in Table 1).

To assess children’s perceived similarity to the protagonist (Hypothesis 3), we asked one question per scenario. For the first scenario (frequency of contributions), the experimenter asked, “How about you? Do you think the teacher calls on you more often, as often as, or less often than the other children?” If children chose “more often” or “less often,” the experimenter then followed up: “Does the teacher call on you a little [more often/less often] or a lot [more often/less often] than the other children?” Answers ranged from 1 (*a lot less often than the other children*) to 5 (*a lot more often than the other children*), with a midpoint of 3 (*as often as the other children*). For the second scenario (length of contributions), children were asked: “How about you? Do you think you talk longer, as long as, or less long than the other children when the teacher asks you a question?” If children chose “longer” or “less long,” then the experimenter followed up: “Do you talk a little [longer/less long] or a lot [longer/less long] than the other children?” Answers ranged from 1 (*a lot less long than the other children*) to 5 (*a lot longer than the other children*), with a midpoint of 3 (*as long as the other children*).

To assess the perceived similarity of classmates to the protagonist (Hypothesis 4), we asked participants if there was a child in their class who behaved like the protagonist in each scenario. For the first scenario (frequency of contributions), children were asked: “Can you tell me if there is a child in your class who is called on by your teacher more often than the other children?” For the second scenario (length of contributions), children were asked: “Can you tell me if there is a child in your class who talks longer than the other children when your teacher asks them a question?”

#### Results: open-ended explanations

We compared the frequency of inherent and extrinsic explanations for each scenario using goodness-of-fit *χ*² tests. These and all other analyses were performed in R (version 4.0.3); some analyses used the *jmv* package (version 2.3.4^39^).

**Fig. 1:**
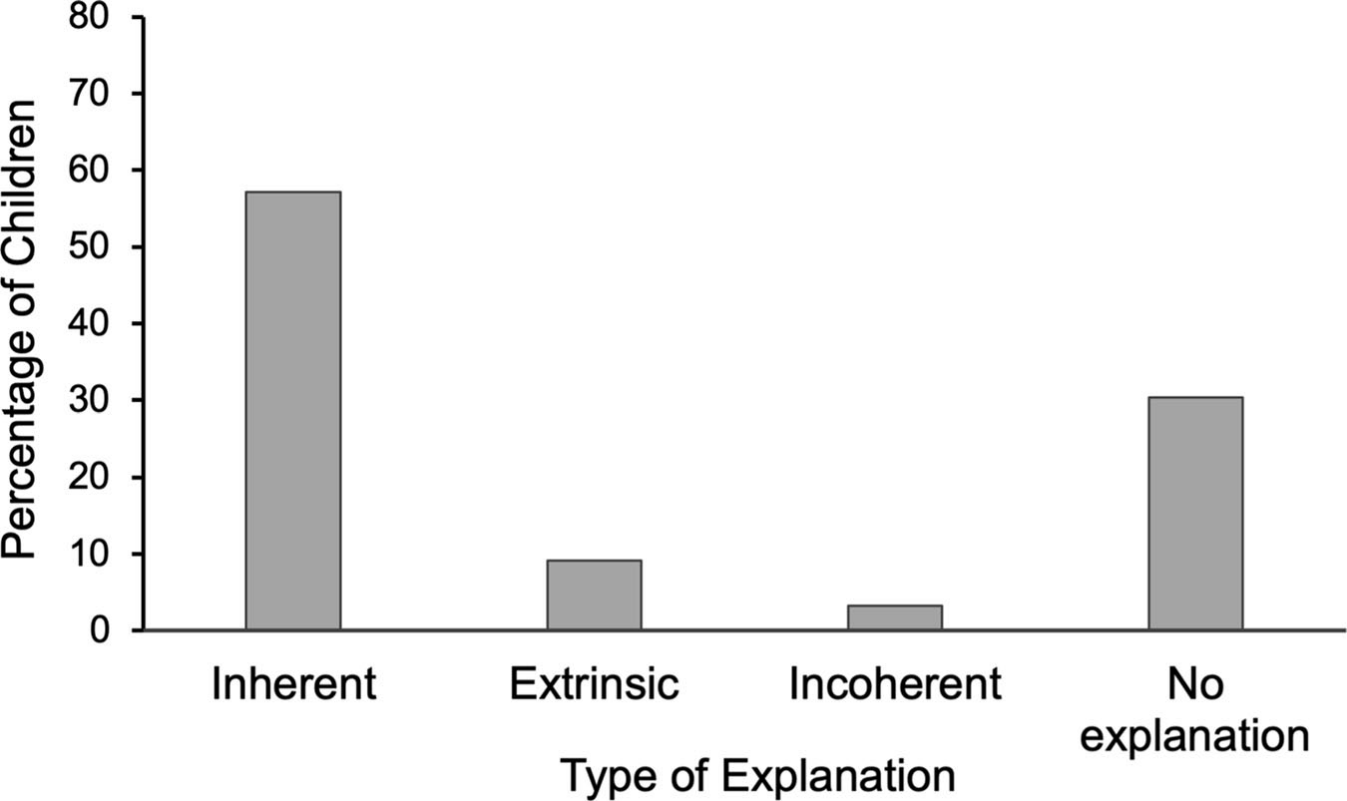
Percentage of each type of explanation for a peer’s frequent contributions in Study 1 (Scenario 1).

We first discuss the results for Scenario 1 (frequency of contributions). The frequency of each type of explanation (i.e., inherent, extrinsic, incoherent/irrelevant, no explanation) is depicted in Fig. 1. In line with Hypothesis 1, children used more inherent than extrinsic factors to explain why a peer made frequent contributions to classroom discussions, *χ*² (1, *N* = 203) = 106.45, *p* < 0.001. Participants’ social class did not predict the content of their explanations (*p* = 0.71), and neither did their gender (*p* = 0.93).

**Fig. 2:**
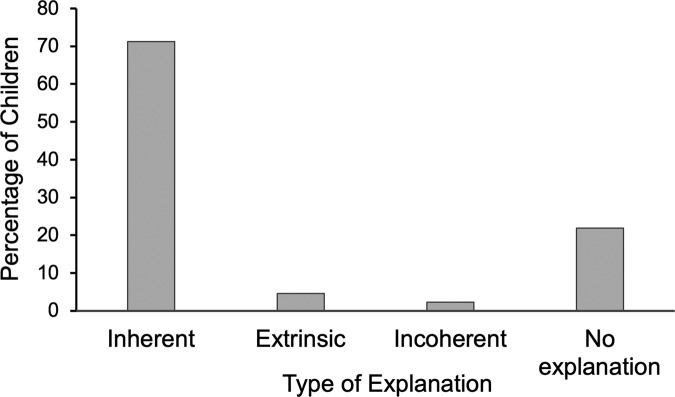
Percentage of each type of explanation for a peer’s lengthy contributions in Study 1 (Scenario 2).

Next, we discuss the results for Scenario 2 (length of contributions). The frequency of each type of explanation (i.e., inherent, extrinsic, incoherent/irrelevant, no explanation) is depicted in Fig. 2. In line with Hypothesis 1, children used more inherent than extrinsic factors to explain why a peer made lengthy contributions to classroom discussions, *χ*² (1, *N* = 232) = 179.38, *p* < 0.001. Participants’ social class did not predict the content of their explanations (*p* = 0.52), and neither did their gender (*p* = 0.71).

#### Results: competence and warmth evaluations

Children’s mean competence and warmth ratings were compared to the scale’s midpoint (i.e., 3) using two-tailed one-sample *t* tests. As evaluations of the fictional protagonist were elicited *relative* to other children in class, this comparison against the midpoint—which was explicitly labeled as indicating that the protagonist was the same as their peers on the relevant dimension—reveals whether participating children evaluated the protagonist as being above, below, or no different than the average child in competence and warmth.

We first discuss the results for Scenario 1 (frequency of contributions). Descriptive statistics are presented in Table 2. In line with Hypothesis 2, children judged the peer who made frequent contributions as being more intelligent (*M* = 3.61, *t* = 8.03, *p* < 0.001, *d* = 0.46, 95% CI = [0.34, 0.58]), as having higher academic achievement (*M* = 3.58, *t* = 7.93, *p* < 0.001, *d* = 0.45, 95% CI = [0.34, 0.57]), as being nicer (*M* = 3.59, *t* = 7.85, *p* < .001, *d* = 0.45, 95% CI = [0.33, 0.57]), and as being more liked by the teacher (*M* = 3.51, *t* = 7.35, *p* < 0.001, *d* = 0.42, 95% CI = [0.30, 0.54]) compared to other children in their class.

**Table 2:** Mean (SD) scores across the seven items in Scenarios 1 (frequency) and 2 (duration) in Study 1

Scenario	1. Is intelligent	2. Is good at school	3. Is nice	4. Liked by the teacher	5. Is bad	6. Is stupid	7. Likes strawberries
Frequency	3.61 (1.33)	3.58 (1.27)	3.59 (1.32)	3.51 (1.22)	2.37 (1.31)	2.46 (1.37)	3.57 (1.37)
Duration	3.27 (1.38)	3.27 (1.35)	3.30 (1.38)	3.18 (1.33)	2.52 (1.41)	2.65 (1.34)	3.26 (1.40)

All ratings were different from the midpoint of the scale (3), *p*s ≤ 0.001 on two-tailed one-sample *t* tests.

Addressing potential low-level response biases, the mean scores for the “bad” and “stupid” items were significantly *below* the midpoint (*M* = 2.37, *t* = −8.45, *p* < 0.001, *d* = −0.48, 95% CI = [−0.60, −0.36], and *M* = 2.46, *t* = −6.81, *p* < 0.001, *d* = −0.39, 95% CI = [−0.51, −0.27], respectively). However, the mean score for the unrelated question about liking strawberries was significantly higher than the midpoint (*M* = 3.57, *t* = 7.24, *p* < 0.001, *d* = 0.41, 95% CI = [0.30, 0.53]), contrary to Hypothesis 5 (see Table 1). One possibility is that the children perceived liking strawberries as something positive and attributed this positive attribute to the protagonist as well (akin to a halo effect, which is not uncommon in developmental research^40,41^).

Next, we discuss the results for Scenario 2 (length of contributions). Similarly to Scenario 1 and consistent with Hypothesis 2, the peer who made lengthy contributions was judged as being more intelligent (*M* = 3.27, *t* = 3.37, *p* < 0.001, *d* = 0.19, 95% CI = [0.08, 0.31]), as having higher academic achievement (*M* = 3.27, *t* = 3.52, *p* < 0.001, *d* = 0.20, 95% CI = [0.09, 0.31]), as being nicer (*M* = 3.30, *t* = 3.78, *p* < 0.001, *d* = 0.22, 95% CI = [0.10, 0.33]), and as being more liked by the teacher (*M* = 3.18, *t* = 2.35, *p* = 0.019, *d* = 0.13, 95% CI = [0.02, 0.25]) than other children.

Contradicting the possibility of low-level biases, the mean scores for the “bad” and “stupid” items were significantly below the midpoint (*M* = 2.52, *t* = −5.87, *p* < 0.001, *d* = −0.34, 95% CI = [−0.45, −0.22], and *M* = 2.65, *t* = −4.54, *p* < 0.001, *d* = −0.26, 95% CI = [−0.37, −0.14], respectively). However, as in Scenario 1 and contrary to Hypothesis 5, the mean score for the control question about liking strawberries was significantly higher than the midpoint (*M* = 3.26, *t* = 3.26, *p* = 0.001, *d* = 0.19, 95% CI = [0.07, 0.30]).

The halo-like effect on the “strawberry” item suggests that children’s answers to the critical competence and warmth questions may have been, in part, the byproduct of low-level response biases, contrary to Hypothesis 5. To explore this possibility further, we adapted a regression-based strategy from Greenwald and colleagues^42^ to test if there is any evidence of above-the-midpoint competence and warmth evaluations *after response biases (as measured by the “strawberry” item) are taken into account*. Each competence and warmth item was regressed on the “strawberry” item. Critically, both the outcome and the predictor (i.e., the “strawberry” item) in each of these models were centered on the midpoint of the scale (i.e., 3 = the protagonist was average—just like everyone else—in their competence and warmth). To accomplish this centering, the number 3 was subtracted from each observation. In regression models with this structure, whether the intercept is significantly greater than zero indicates whether children thought that the protagonist was more competent and warmer than the average child, even after adjusting for the response bias captured by the “strawberry” item. Intercepts were positive and significant for all competence and warmth judgments in Scenario 1 (*p*s < 0.001) and for three of the four competence and warmth judgments in Scenario 2 (*p*s < 0.031). The only exception was the intercept of the “liked by the teacher” item in Scenario 2 (*b* = 0.09, *SE* = 0.07, *p* = 0.24). These results suggest that children’s positive view of the competence and warmth of peers who make substantial contributions to class discussions cannot be explained by a low-level response bias, consistent with the spirit of Hypothesis 5.

#### Results: participants’ perceived similarity with the protagonist as a function of participants’ social class

An ancillary hypothesis was that working-class (vs. middle- and upper-class) children would see themselves as less similar to the protagonists in the vignettes (see Hypothesis 3 in Table 1). To compare how similar working-class versus middle- and upper-class children perceived themselves to be to the protagonist in each scenario, we used independent-sample *t* tests. The children were categorized as coming from a working-class versus middle- and upper-class background using the median split criterion described above.

We first discuss the results for Scenario 1 (frequency of contributions). Contrary to Hypothesis 3, working-class children (*M* = 2.96, *SD* = 1.14) and middle- and upper-class children (*M* = 2.88, *SD* = 1.15) did not differ significantly in how similar they perceived themselves to be to the protagonist, *t* = 0.57, *p* = 0.57, *d* = 0.07, 95% CI = [−0.16, 0.29].

Next, we discuss the results for Scenario 2 (length of contributions). Contrary to Hypothesis 3, working-class children (*M* = 2.87, *SD* = 1.15) and middle- and upper-class children (*M* = 2.91, *SD* = 1.08) did not differ significantly in how similar they perceived themselves to be to the protagonist, *t* = −0.29, *p* = 0.77, *d* = −0.03, 95% CI = [−0.26, 0.19].

#### Results: perceived similarity of classmates with the protagonist as a function of classmates’ social class

Another ancillary hypothesis was that children would name working-class (vs. middle- and upper-class) classmates less often as being similar to the protagonists in the vignettes (see Hypothesis 4 in Table 1). To compare the number of working-class versus middle- and upper-class classmates identified as similar to the protagonists, we used goodness-of-fit *χ*² tests. The classmates named by the participants were categorized as coming from a working-class versus middle- and upper-class background using the median split criterion described above.

We first discuss the results for Scenario 1 (frequency of contributions). Only 93 of the participating children (30.4%) identified a classmate in response to our prompt for Scenario 1. In line with Hypothesis 4, working-class (vs. middle- and upper-class) classmates were significantly less likely to be identified as being frequently called on by the teacher, *χ*² (1, *N* = 93) = 5.68, *p* = 0.017. Of the classmates identified by our participants as similar to the protagonist, 37.6% were from working-class families (i.e., below the median Social Position Index), and 62.4% were from middle- and upper-class families (i.e., above the median Social Position Index).

Next, we discuss the results for Scenario 2 (length of contributions). Only 126 of the participating children (41.2%) identified a classmate in response to our prompt for Scenario 2. Contrary to Hypothesis 4 and the results for Scenario 1, working class (vs. middle- and upper-class) classmates were not significantly less likely to be identified as talking longer, *χ*² (1, *N* = 126) = 0.51, *p* = 0.48. Of the classmates identified by our participants as similar to the protagonist, 46.8% were from working-class families, and 53.2% were from middle- and upper-class families.

To summarize, the present results reveal that a large sample of Grande-Section children in France was more likely to use inherent (vs. extrinsic) factors to explain why a fictional peer contributes more than others to classroom discussions (Hypothesis 1). Similarly, children judged this fictional peer as higher in both competence and warmth than other children (Hypothesis 2). Contrary to ancillary Hypothesis 3, working-class children did not perceive themselves as making fewer substantial contributions to whole-class discussions relative to their middle- and upper-class peers. However, their peers did identify children from working-class backgrounds as contributing less often to whole-class discussions relative to their middle- and upper-class counterparts, consistent with ancillary Hypothesis 4 and with reality^26^. This difference was not present for the length of contributions to whole-class discussions.

From the perspective of cumulative and reproducible science, this preregistered replication of a previous study^26^ on a new, larger sample increases confidence in the claim that children tend to explain differences in achievement in inherent terms, consistent with a broader literature suggesting the presence of an inherence bias in explanation (for a review, see ref. ^7^). It is also noteworthy that the present study featured several methodological improvements that increased confidence in our conclusions. For instance, we included an explicit midpoint that allowed children to indicate that the protagonist of the scenarios was no different than their peers. We also included a set of control items to address the possibility of low-level response biases. While some results (specifically, children’s responses to a control question about whether the protagonist likes strawberries) raised the possibility of halo effects, statistically adjusting for these response biases left our original conclusions largely intact.

### Study 2

In Study 2, we used data from the first wave of an ongoing longitudinal study to replicate and extend the findings of Study 1. Specifically, the present study addressed several limitations of Study 1. First, Study 1 did not include scenarios that describe children who contribute less than average to class discussions—a pattern that is more typical of working-class children. We addressed this limitation in the present study by designing two new scenarios in which children were asked to imagine doing better and—critically—doing worse than a peer on a math exercise. Second, Study 1 focused exclusively on differences in participation in whole-class discussions. This focus was motivated by the fact that (a) whole-class discussions take up a significant amount of time in preschool (they occur several times a day) and (b) oral participation is a core aspect of the preschool curriculum in France. Nevertheless, by asking children to explain differences in *math* achievement in the present study, we were able to examine whether the inherence bias in children’s explanations generalizes to other aspects of achievement. Third, in Study 1, we asked children to explain the behavior of fictional peers. In the present study, we tested whether the same explanatory bias would be found when children are asked to explain differences in achievement between themselves and their peers. Explanations for observed achievement differences involving the *self* may be particularly influential, given their potential downstream consequences for children’s self-concepts and self-efficacy. Fourth, and related to the preceding point, the structure of the scenarios in the present study enabled us to vary the specific syntax we used to elicit children’s explanations (relative to Study 1) and thus further assess the robustness and generalizability of children’s inherence bias.

Participants were 304 preschoolers (enrolled with administrative authorization and written parental consent) from 25 classrooms of Grande-Section in the French preschool system (144 girls, 160 boys; *M*_age_ = 5.7 years, range = 5.05 years to 6.7 years). This sample was recruited from the same region of France as the sample for Study 1 and, like this previous sample, was similar to broader French society in terms of social class (*M* = 104, *SD* = 29.2). A sensitivity analysis suggested that we had 80% power to detect effects of magnitude *w* ≥ 0.16 on a *χ²* test and *d* ≥ 0.16 on a one-sample *t* test.

Children were tested individually in a quiet room next to their classroom. The sessions lasted about 10 minutes and were recorded and later transcribed. All the questions were verbally administered by the experimenter. Children were prompted to provide explanations for two different scenarios: a scenario involving downward comparison (in which the participating child was said to do better than a peer) and a scenario involving upward comparison (in which the participating child was said to do worse than a peer). The downward comparison (success) item was presented first, followed by the upward comparison (failure) item. This fixed order was designed to minimize children’s negative affect. The stimuli were constructed and delivered with PsychoPy (version 2021.2.3^43^).

#### Measure: open-ended explanations

Children were asked to imagine two scenarios in which they did better and, separately, worse than a hypothetical same-gender peer on a math exercise (see this project’s Open Science Framework repository, https://osf.io/w9sur/, for the actual exercises):

1. *Downward comparison*: “Imagine that today the teacher introduces an exercise like this one to the class. Imagine that you do better than one of your classmates. How would you explain the fact that you did better than the other child?”
2. *Upward comparison*: “Imagine that today the teacher introduces an exercise like this one to the class. Imagine that you do less well than one of your classmates. How would you explain the fact that you did less well than the other child?”

Children’s open-ended explanations for downward and upward comparisons were coded independently by two researchers. The “inherent” explanation code (e.g., “Because I am intelligent,” “Because I wasn’t paying attention”) and the “extrinsic” explanation code (e.g., “Because it is too hard,” “Because they bother me. They make too much noise. Because sometimes they talk too loud”) were not mutually exclusive in this study; thus, a participant’s response could be coded as both or neither (see ref. ^44^ for a similar coding procedure). We switched to this type of coding because children tended to produce longer and more complex responses than in Study 1. Inter-rater reliability was high, and disagreements were resolved through discussion:

- Scenario 1 (Downward)—Inherent: 99% agreement, Cohen’s κ = 0.94;
- Scenario 1 (Downward)—Extrinsic: 97% agreement, Cohen’s κ = 0.90;
- Scenario 2 (Upward)—Inherent: 96% agreement, Cohen’s κ = 0.84;
- Scenario 2 (Upward)—Extrinsic: 95% agreement, Cohen’s κ = 0.84.

We note that the rate of non-responses in this study was higher than in Study 1: 41.1% for Scenario 1 (Downward Comparison) and 37.2% for Scenario 2 (Upward Comparison). Informal conversations with teachers from the classrooms where we recruited children suggested that the practice of providing comparative feedback was not common in those classrooms, which may have made it difficult for children to provide explanations. Additionally, this type of comparative information is also less common than being exposed to differences in oral participation, which could explain the larger proportion of non-responses here compared with Study 1.

#### Results: open-ended explanations

**Fig. 3:**
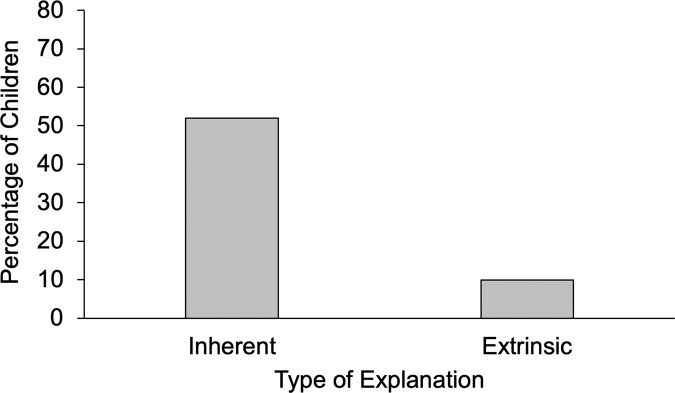
Percentage of children who mentioned inherent vs. extrinsic factors when explaining the downward comparison situation in Study 2 (Scenario 1).

**Fig. 4:**
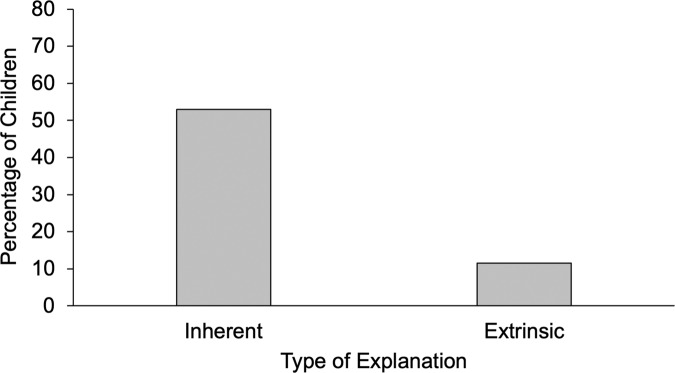
Percentage of children who mentioned inherent vs. extrinsic factors when explaining the upward comparison situation in Study 2 (Scenario 2).

We used McNemar’s tests to compare the frequencies with which children used inherent and extrinsic factors in their explanations. (Goodness-of-fit *χ*² tests are no longer appropriate when inherent and extrinsic explanations are coded in a non-mutually-exclusive fashion.) The percentage of children who mentioned inherent versus extrinsic factors is depicted in Figs. 3 and 4.

We first discuss the results for Scenario 1 (downward comparison). In line with our predictions, children were more likely to mention inherent (*n* = 158) than extrinsic (*n* = 30) factors to explain why they were more successful than one of their peers, McNemar *χ*² (1) = 103.39, *p* < 0.001 (see Fig. 3). Participants’ social class did not predict their likelihood of generating an inherent or an extrinsic explanation (*p*s > 0.18). Girls did not generate more extrinsic explanations than boys (*p* = 0.86), but they generated more inherent explanations (*p* = 0.032). As this difference was not anticipated on a priori grounds, we do not interpret it further.

Next, we discuss the results for Scenario 2 (upward comparison). In line with our predictions, children were more likely to mention inherent (*n* = 161) than extrinsic (*n* = 35) factors to explain why they were less successful than one of their peers, McNemar *χ*² (1) = 94.13, *p* < 0.001 (see Fig. 4). Participants’ social class did not predict their likelihood of generating an inherent or an extrinsic explanation (*p*s > 0.35). Gender was not predictive either (*p*s > 0.86).

To summarize, the findings of Study 2 suggest, again, that preschoolers tend to explain differences in achievement as being due to inherent rather than extrinsic factors. Importantly, this study extends Study 1 in several ways. It documents the existence of an inherence bias in children’s explanations (a) for positive and negative achievement outcomes; (b) for differences in achievement more broadly, beyond differences of oral participation; (c) for differences between children’s own achievement outcomes and those of others; and (d) with a range of syntactic frames for the “why?” question.

## Discussion

Taken together, the present studies suggest that preschool children are more likely to use inherent (vs. extrinsic) factors to explain differences in academic achievement. By replicating and extending the results of a previous study^26^ on new and larger samples and with different stimuli, the present work increases confidence in the argument that children exhibit an inherence bias when explaining differences in achievement.

An important limitation of this work is that we did not examine the consequences of children’s explanations. However, extensive evidence in the literature highlights that children’s explanations have consequences for their motivation, engagement, and academic performance (^22,45–48^; see ref. ^3^ for a review). A powerful mechanism underlying these effects is that inherent explanations make the experience of being outperformed by peers particularly threatening for children’s self-concepts and self-efficacy, which in turn undermines motivation and achievement. Given that children from marginalized backgrounds, such as working-class or ethnic-minority children, are more likely to experience difficulty and be outperformed by their peers (due, in part, to having less “cultural capital” in school contexts^31^), the inherence bias may ultimately amplify group inequalities in the classroom.

Study 1 revealed no evidence that children from middle- and upper-class backgrounds perceive themselves as contributing to whole-class discussions more than children from working-class backgrounds. This is surprising because prior evidence suggested that substantial social class differences do exist in this respect^26^. One possibility is that the social class differences in participation that have been documented in prior samples were absent in the sample recruited for Study 1. Another possibility, which we believe to be more likely, is that children’s self-reports did not accurately track the participation differences that were present in their classrooms. It may be, for example, that when children from middle- and upper-class families answer questions about whether the teacher calls on them more or less often than on other children, they compare themselves to other children who raise their hands for an opportunity to speak, who are also likely to be from middle- and upper-class families, which could then lead them to underestimate how often they are called on relative to the average child in their class. The fact that children from middle- and upper-class (vs. working-class) backgrounds were identified by their peers as contributing more frequently to whole-class discussions is consistent with this possibility. More research is needed to uncover the precise reasons why children’s self-reported participation does not exhibit the social class differences that their actual participation does.

To conclude, the present studies—as well as the Goudeau et al. ^26^ study they replicated and extended—provided robust evidence for an inherence bias in children’s explanations for differences in achievement, thereby contributing to theory on the early construction of inequalities in education. As students from middle- and upper-class families contribute more to class discussions and generally perform better academically than working-class children starting from the earliest grades, this bias in explanation is likely to lead children (and, possibly, teachers as well) to perceive these students as smarter and more socially skilled than their working-class peers, making social class achievement differences seem “natural”, legitimate, and thus difficult to challenge.

## Methods

### Ethics approval

Studies 1 and 2 were approved by the Ethics Committee for Human Research of the Universities of Tours and Poitiers (CER-TP, n°2021-10-01). All aspects of these studies were conducted in accordance with the ethical standards of the Declaration of Helsinki.

### Open and transparent scientific practices

The pre-registration for Study 1 and the data and R scripts for both studies are available on the Open Science Framework: https://osf.io/w9sur/ We disclose two deviations from the preregistration in Study 1. First, the question asking children to identify a classmate who is similar to the protagonist was also followed up by asking whether this classmate spoke “a little more” vs. “a lot more.” This question did not elicit much variability and was not directly relevant to our hypotheses, so we did not analyze the corresponding data. Second, the preregistration mistakenly lists follow-up questions in the negative direction as well (“a little less” vs. “a lot less”), but such questions were not actually administered.

### Reporting summary

Further information on research design is available in the Nature Research Reporting Summary linked to this article.

### Supplementary information

Reporting summary

## Data Availability

The pre-registration for Study 1 and the data for both studies are available on OSF: https://osf.io/w9sur/.

## Code availability

The complete R script is available on OSF: https://osf.io/w9sur/.
