# Supplementary material for: The inherence bias in preschoolers’ explanations for achievement differences: replication and extension
Source: NPJ Sci Learn. 2024 Feb 20;9:10. doi: 10.1038/s41539-024-00218-w (PMC10879106; doi:10.1038/s41539-024-00218-w)
Supplement: Supplementary file 1 — Reporting summary [file 41539_2024_218_MOESM1_ESM.pdf]

nature portfolio

Corresponding author(s): Sébastien Goudeau

Last updated by author(s): 27/09/2023

Reporting Summary

Nature Portfolio wishes to improve the reproducibility of the work that we publish. This form provides structure for consistency and transparency in reporting. For further information on Nature Portfolio policies, see our Editorial Policies and the Editorial Policy Checklist.

Statistics

For all statistical analyses, confirm that the following items are present in the figure legend, table legend, main text, or Methods section.

n/a	Confirmed	
☐	☒	The exact sample size (*n*) for each experimental group/condition, given as a discrete number and unit of measurement
☐	☒	A statement on whether measurements were taken from distinct samples or whether the same sample was measured repeatedly
☐	☒	The statistical test(s) used AND whether they are one- or two-sided<br>*Only common tests should be described solely by name; describe more complex techniques in the Methods section.*
☒	☐	A description of all covariates tested
☒	☐	A description of any assumptions or corrections, such as tests of normality and adjustment for multiple comparisons
☐	☒	A full description of the statistical parameters including central tendency (e.g. means) or other basic estimates (e.g. regression coefficient) AND variation (e.g. standard deviation) or associated estimates of uncertainty (e.g. confidence intervals)
☐	☒	For null hypothesis testing, the test statistic (e.g. *F*, *t*, *r*) with confidence intervals, effect sizes, degrees of freedom and *P* value noted<br>*Give P values as exact values whenever suitable.*
☒	☐	For Bayesian analysis, information on the choice of priors and Markov chain Monte Carlo settings
☒	☐	For hierarchical and complex designs, identification of the appropriate level for tests and full reporting of outcomes
☐	☐	Estimates of effect sizes (e.g. Cohen's *d*, Pearson's *r*), indicating how they were calculated

Our web collection on statistics for biologists contains articles on many of the points above.

Software and code

Policy information about availability of computer code

Data collection	*Provide a description of all commercial, open source and custom code used to collect the data in this study, specifying the version used OR state that no software was used.*
Data analysis	Analyses were performed in R (version 4.0.3); some analyses used the jmv package (version 2.3.4; Selker et al., 2022).

For manuscripts utilizing custom algorithms or software that are central to the research but not yet described in published literature, software must be made available to editors and reviewers. We strongly encourage code deposition in a community repository (e.g. GitHub). See the Nature Portfolio guidelines for submitting code & software for further information.

Data

Policy information about availability of data

All manuscripts must include a data availability statement. This statement should provide the following information, where applicable:

- Accession codes, unique identifiers, or web links for publicly available datasets
- A description of any restrictions on data availability
- For clinical datasets or third party data, please ensure that the statement adheres to our policy

the data for both studies, and the complete R script are available on OSF: https://osf.io/w9sur/?view_only=d2970fb7d56944ea97f3f62ff4ba1e07

Research involving human participants, their data, or biological material

Policy information about studies with human participants or human data. See also policy information about sex, gender (identity/presentation), and sexual orientation and race, ethnicity and racism.

Reporting on sex and gender	Gender was collected in order to be able to describe our sample. We did not have specific hypothesis regarding gender but test the potential effect of gender on reviewer' request. Data concerning gender is available on OSF..
Reporting on race, ethnicity, or other socially relevant groupings	We used the Social Position Index as a proxy for socioeconomic status (SES; Rocher, 2016). This indicator is a standardized continuous variable, with a mean of 100 and a standard deviation of 30. It has been developed on large French databases in order to capture multiple dimensions linked to socioeconomic status (e.g., educational attainment, parental education, material conditions, cultural capital). We collected information about the occupation of each parent and then assigned a Social Position Index value to each child based on these occupations (Rocher, 2016). The composition of our studies' sample in terms of socioeconomic status was similar to the social structure of the French society. In study 1, we had specific pre-registered hypotheses that we tested.
Population characteristics	In study 1, Participants included 306 preschoolers (enrolled with administrative authorization and written parental consent) from 27 classrooms of Grande-Section, the last year in French preschool, roughly equivalent to kindergarten in the U.S. and other countries (142 girls, 164 boys; Mage = 5.6 years, range = 4.9–6.6).<br><br>In study 2, participants were 304 preschoolers (enrolled with administrative authorization and written parental consent) from 25 classrooms of Grande-Section in the French preschool system (144 girls, 160 boys; Mage = 5.7 years, range = 5.05 years to 6.7 years).
Recruitment	We recruited participants through teachers' preschool with parental informed consent
Ethics oversight	Ethics Committee for Human Research of the Universities of Tours and Poitiers (CER-TP, n°2021-10-01).

Note that full information on the approval of the study protocol must also be provided in the manuscript.

Field-specific reporting

Please select the one below that is the best fit for your research. If you are not sure, read the appropriate sections before making your selection.

☐ Life sciences ☒ Behavioural & social sciences ☐ Ecological, evolutionary & environmental sciences

For a reference copy of the document with all sections, see nature.com/documents/nr-reporting-summary-flat.pdf

Behavioural & social sciences study design

All studies must disclose on these points even when the disclosure is negative.

Study description	data were quantitative and collected through individual interview.
Research sample	In study 1, Participants included 306 preschoolers (enrolled with administrative authorization and written parental consent) from 27 classrooms of Grande-Section, the last year in French preschool, roughly equivalent to kindergarten in the U.S. and other countries (142 girls, 164 boys; Mage = 5.6 years, range = 4.9–6.6).<br>In study 2, participants were 304 preschoolers (enrolled with administrative authorization and written parental consent) from 25 classrooms of Grande-Section in the French preschool system (144 girls, 160 boys; Mage = 5.7 years, range = 5.05 years to 6.7 years).<br>The composition of our studies' sample in terms of socioeconomic status was similar to the social structure of the French society.
Sampling strategy	For study 1, following a power analysis based on the effect size from the original study (Goudeau et al., 2023), we preregistered a sample of 200 children. To compensate for potential logistical issues caused by the COVID crisis, we increased the number of classrooms we contacted. Unexpectedly high rates of interest from teachers and parents resulted in a final sample of 306 participants. A sensitivity analysis using this larger sample indicated that we had 80% power to detect effects of magnitude $w \geq 0.16$ on a χ^2 test and $d \geq 0.16$ on a one-sample t test.<br>For study 2, the sample was recruited from the same region of France as the sample for Study 1 and, like this previous sample, was similar to broader French society in terms of socioeconomic status (M = 104, SD = 29.2). A sensitivity analysis suggested that we had 80% power to detect effects of magnitude $w \geq 0.16$ on a χ^2 test and $d \geq 0.16$ on a one-sample t test
Data collection	Children were tested individually in a quiet room next to their classroom. The sessions lasted around 10 minutes and were recorded and later transcribed. The experimenter was aware about the hypotheses.
Timing	study 1: december 2021-May 2022.<br>study 2: december 2022-May 2022
Data exclusions	no data exclusion
Non-participation	no participants dropped out during the data collection.

Randomization	NA

Reporting for specific materials, systems and methods

We require information from authors about some types of materials, experimental systems and methods used in many studies. Here, indicate whether each material, system or method listed is relevant to your study. If you are not sure if a list item applies to your research, read the appropriate section before selecting a response.

Materials & experimental systems

n/a	Involved in the study
☒	☐ Antibodies
☒	☐ Eukaryotic cell lines
☒	☐ Palaeontology and archaeology
☒	☐ Animals and other organisms
☒	☐ Clinical data
☒	☐ Dual use research of concern
☒	☐ Plants

Methods

n/a	Involved in the study
☒	☐ ChIP-seq
☒	☐ Flow cytometry
☒	☐ MRI-based neuroimaging
